# P-1440. Are Veterans Willing to Complete Sexual Health Screening In Primary Care?: Lessons Learned from a Population Level Sexual Health Questionnaire

**DOI:** 10.1093/ofid/ofae631.1613

**Published:** 2025-01-29

**Authors:** Minh Q Ho, Puja Van Epps, Brigid Wilson, Karen Slazinski, Linda Chia, Matthew Cole, Nicholas Scapelito, Katrina Mclean, Mohammed Ahmed

**Affiliations:** Orlando VA Healthcare System, Orlando, Florida; Veterans Health Administration, Case Western Reserve University School of Medicine, Cleveland, Ohio; VA Northeast Ohio Healthcare System, Cleveland, Ohio; Orlando VA Healthcare System, Orlando, Florida; VA, Bellevue, Washington; VA Capital Health Care Network (VISN 5), Veterans Health Administration, Huntsville, Alabama; Orlando VAHCS, Kissimmee, Florida; Orlando VAHCS, Kissimmee, Florida; Orlando VA Medical Center, Orlando, Florida

## Abstract

**Background:**

The CDC recommends routine sexual histories (SH), yet they are rarely completed in primary care (PC) due to barriers ranging from patient discomfort to provider time constraints. We implemented a system wide nurse-led sexual health screening tool at a large VA hospital. Here we report on patient acceptability of sexual histories and sexual behaviors of Veterans at a population level.

**Table 1:**
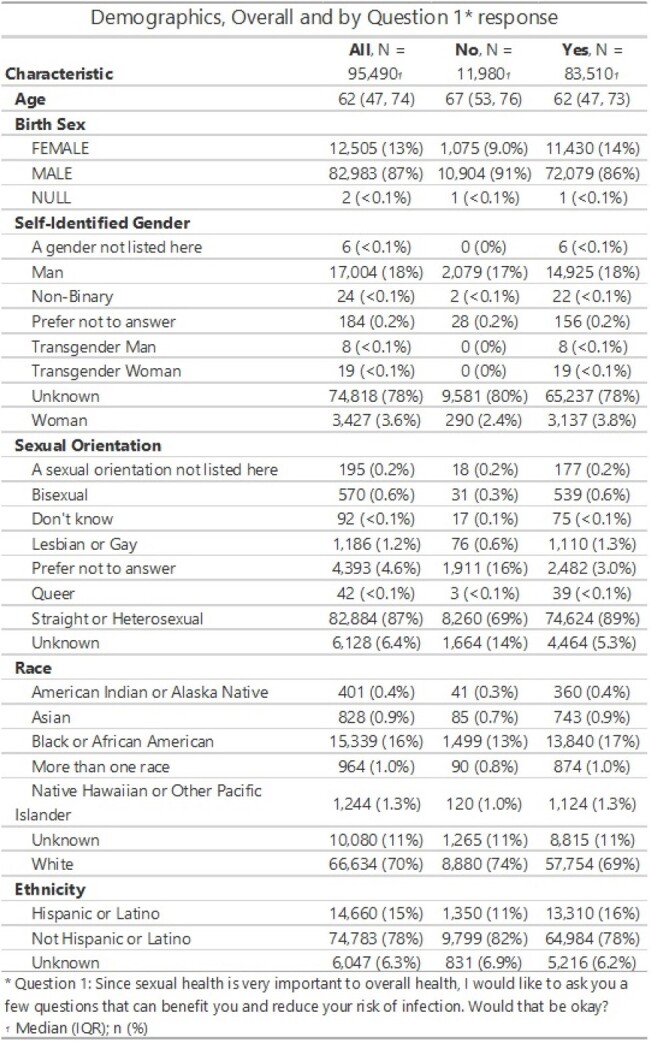
Demographics, Overall by Question 1 Response This table describes demographics of those who agreed to discuss their sexual health, ie said Yes to Question 1

**Methods:**

We deployed a short SH screening questionnaire to be conducted during PC visits. Administered mostly by nurses, the questionnaire began with a consent question. Those with an affirmative response proceeded to answer additional questions. We queried the VA’s electronic database to ascertain patient demographics including age, birth sex, sexual orientation, gender identity, race/ethnicity, Rural-Urban Commuting Area codes. We describe the population as medians and proportions.

**Table 2:**
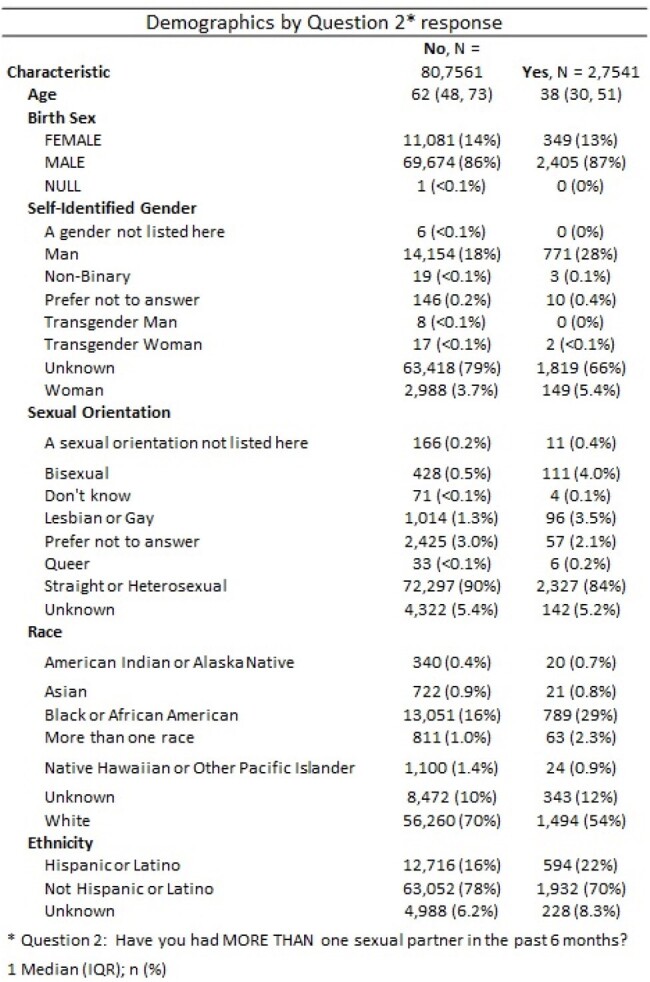
Demographics, Overall by Question 2 Response This table describes demographics of those who agreed to discuss about sexual health and have more than one sexual partner, ie said Yes to Question 2

**Results:**

Over 2-years (April 14, 2022-24) 130,986 SH screening questionnaires were offered to 95,490 unique Veterans, with 83,510 (87%) agreed at least once (Table 1). Birth sex females were more likely than males to agree to SH (91% vs. 87%). Most 65 and older agreed to the screening (85%). Supermajority of Veterans who have recorded a sexual identity as LGBQ+ agreed to SH (1688/1798, 94%). Similarly, 96% of those who have recorded self-identified gender as transgender man, transgender woman, or non-binary also agreed to SH. Racial or ethnic minority patients were more likely to agree to SH than Whites: 90% Black vs. 87% White; 91% vs. 87% non-Hispanic. Of 83,510 patients who agreed, 2754 (3%) had more than 1 sexual partner in the last 6 months, with younger age, LGBQ+ identity, Black race and Hispanic ethnicity associated with likelihood of more than 1 sexual partner (Table 2). Of these, 4% reported sexual partners of more than 1 gender, and 21% of this subset had a self-described sexual orientation as straight or heterosexual.

Sexual Health Questions

**Figure:**
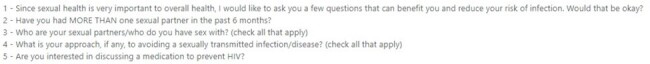

**Conclusion:**

A brief SH screening questionnaire administered by nurses in PC settings is feasible and highly acceptable to patients. Patients who identify as minority racial, ethnic, sexual or gender identity groups are very willing to provide SH, in some cases at much higher rates than non-minority peers. Other healthcare systems can implement similar nurse-led screening tools to improve SH taking in PC settings

Age Breakdown for Question 1

**Figure:**
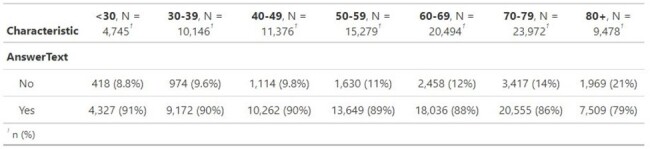

This table describes the age breakdown for those who said Yes to Question 1, indicating both young and old population agree to discuss about sex.

**Disclosures:**

**All Authors**: No reported disclosures

